# “I have to do what I believe”: Sudanese women’s beliefs and resistance to hegemonic practices at home and during experiences of maternity care in Canada

**DOI:** 10.1186/1471-2393-13-51

**Published:** 2013-02-25

**Authors:** Gina MA Higginbottom, Jalal Safipour, Zubia Mumtaz, Yvonne Chiu, Patricia Paton, Jennifer Pillay

**Affiliations:** 1 Faculty of Nursing, University of Alberta, Edmonton, Canada; 2 School of Public Health, University of Alberta, Edmonton, Canada; 3 Multicultural Health Brokers Co-operative, Edmonton, AB, Canada; 4 Women’s Health, Alberta Health Services, Edmonton, AB, Canada

**Keywords:** Canada, Sudanese, Beliefs, Culture, Focused ethnography, Maternity, Refugee, Pregnancy

## Abstract

**Background:**

Evidence suggests that immigrant women having different ethnocultural backgrounds than those dominant in the host country have difficulty during their access to and reception of maternity care services, but little knowledge exists on how factors such as ethnic group and cultural beliefs intersect and influence health care access and outcomes. Amongst immigrant populations in Canada, refugee women are one of the most vulnerable groups and pregnant women with immediate needs for health care services may be at higher risk of health problems. This paper describes findings from the qualitative dimension of a mixed-methodological study.

**Methods:**

A focused ethnographic approach was conducted in 2010 with Sudanese women living in an urban Canadian city. Focus group interviews were conducted to map out the experiences of these women in maternity care, particularly with respect to the challenges faced when attempting to use health care services.

**Results:**

Twelve women (mean age 36.6 yrs) having experience using maternity services in Canada within the past two years participated. The findings revealed that there are many beliefs that impact upon behaviours and perceptions during the perinatal period. Traditionally, the women mostly avoid anything that they believe could harm themselves or their babies. Pregnancy and delivery were strongly believed to be natural events without need for special attention or intervention. Furthermore, the sub-Saharan culture supports the dominance of the family by males and the ideology of patriarchy. Pregnancy and birth are events reflecting a certain empowerment for women, and the women tend to exert control in ways that may or may not be respected by their husbands. Individual choices are often made to foster self and outward-perceptions of managing one’s affairs with strength.

**Conclusion:**

In today’s multicultural society there is a strong need to avert misunderstandings, and perhaps harm, through facilitating cultural awareness and competency of care rather than misinterpretations of resistance to care.

## Background

As a consequence of globalization and various conflicts throughout the world, population movement and immigration is ongoing and contributes to increasing diversity within any given society [1,2]. Immigration and the associated processes of settlement, integration and acculturation, can be stressful threats to individuals’ health and wellbeing, ultimately influencing social integration and social cohesion [3].

Numerous studies have focused on the health of newcomer, immigrant and refugee populations in Canada [4-7]. Amongst the literature, it is evident that refugee women are one of the most vulnerable groups [8]. Moreover, the perinatal period may place newcomer women, and their infants, at a higher risk for poor health outcomes due to their immediate and acute needs for accessing health care services during which they may encounter difficulties [9,10]. A challenge when conducting research to gain understandings and devise health promotion strategies particularly for refugee populations, is to appreciate that there is large heterogeneity *within* the refugee population resulting from various differences in ethnocultural orientation, values, and pre-migrant experiences which may have been burdened with war or conflict. Often divisions and factions are played out in social dynamics in the new receiving community [11].

### Maternity care in Canada

Within the Canadian context, maternity care is configured at the provincial level however all provinces and territories are legally required to comply with the Canada Health Act of 1984. Whilst some variation exists between provinces in respect of service constellation, national care standards exist and public funding of the healthcare system covers the costs of most maternity care services. Almost all births (98%) are attended by physicians, most commonly obstetricians but also family physicians [12]. Midwives provide only 3% and 2.5% of prenatal and hospital delivery care respectively [12]. In recent decades greater midwifery service provision has evolved although midwives remain relatively few in number, partly due to the profession having been regulated first in 1992 in Ontario, only currently regulated in 9 of the 13 provinces and territories, and with services only funded through six provincial and territorial health plans [13]. Reports contain highlights of the likelihood of declining numbers of obstetricians, although it seems likely that many women would accept care from nurses/midwives and therefore the midwifery contribution to maternity care may increase [12]. Such a shift would align with the guiding principles of family-centered care in which birth is emphasized as a normal health process recognizing the importance of social support, informed choice, and respect [14].

### Newcomer’s maternity experiences and cultural issues

Newcomers are often faced with language barriers, lack of social supports and lack of knowledge about the availability of conventional and alternative health care services. These challenges may place some individuals at greater risk of health problems [15]. The risks seem to be higher among pregnant women whom have special needs with respect to pre- and postnatal care [9,16-19]. Moreover, the phenomenon is complex and multi-faceted with determinants moving beyond medical care into the psychosocial, socioeconomic, sociopolitical, ethnocultural, and citizenship spheres. Due to this complexity, understandings about the maternity experience of immigrant women are not likely to yield to unidisciplinary analysis and explanation.

Available Canadian studies largely demonstrate a lack of cultural competency and sensitivity on the part of health professionals. Of particular concern is that language barriers can create miscommunication and misunderstandings which may threaten a child’s and mother’s health [9,10,20]. Two other factors serving as barriers to seeking health care among immigrant women are weak social support and limited access to culturally and linguistically appropriate information [10,18,21-23].

Limited overall health care accessibility is supported by the results of a recent population survey of women in Canada, which found that fewer immigrant than Canadian-born women attended prenatal classes and more found it difficult to see a provider for their own and their child’s care [18]. Moreover, although the women responding to this survey reported similar satisfaction with the competency, compassion and respect demonstrated when they received health care, fewer immigrant women were contacted by a health provider at home after hospital discharge, which is a usual practice in Canada due to its early postpartum discharge protocols. Apart from accessibility issues, unfortunately not all immigrant women have similarly reported satisfaction with their care; many of 432 Somali women surveyed by Chalmers and Hashi [24] perceived their doctors to be lacking in competence and their nurse to be insensitive to their postpartum pain. A large majority of the women also reported that their providers indicated lack of respect for their cultural practices through verbal and non-verbal expressions of disgust and surprise.

Religious and traditional beliefs may also present as challenges for newcomers when using health care. Beliefs about childbearing practices can be very diverse among women with different sociocultural backgrounds. For instance, women migrating to Western countries may not feel comfortable with the highly medicalized maternity care. Adherence to ethnoculturally specific traditional belief systems learned through socialization processes may create for some women a very different conceptualization of maternity care and health care more generally. They may view the processes of pregnancy and delivery as normal events which do require special attention or concern. Consequently, they may not seek help and maternity care and may not attend available prenatal programs [15,25]. As Carolan [25] revealed, sub-Saharan newcomer African women in Australia are not comfortable with pain relief injections since they believe that pain relief disrupts normal events and that labor pain is associated with immediate birth; using pain relief interrupts the natural birthing process. Another study exploring beliefs of newcomer African women indicated that pain relief was thought to slow down the delivery and to be harmful for the baby (producing strange characteristics) or to cause the baby to be “sleepy” and “drunk” [15]. Considering labour as best left to run its natural course, sub-Saharan African women often offer strong resistance to caesarean sections [15].

Ethnocultural beliefs and customs may also influence nutrition and food practices during and after pregnancy. Based on some traditional practices, after delivery women are not supposed to drink or eat cold water or food, or take a bath [25]. These beliefs seem to be rooted in Islamic, Eastern and traditional Greek medicine, which divides the essence of foods to “cold” and “warm” and whereby the sick person shall avoid anything related to “coldness”. Avoidance of cold allows the person to warm his/her body and to get more energy to overcome the sickness [26].

### Sudanese context

Sudan is located in the northeast of Africa, and following many years of extreme conflict the country recently divided into North Sudan (largely Muslims) and South Sudan (largely Christians). In most sub-Saharan countries, established traditional sociocultural norms have very important roles especially within family matters; the dominant role of the male is strongly supported by the society [27]. In this respect, religious edicts assert a powerful influence on everyday life. Many Sudanese women experience domestic violence from their husbands which represents a significant factor in these women’s pre-migration history [27,28]. Polygamy is common in some parts of Sudan and because of cultural and socioeconomic barriers contraception is not widely available [27]. Central to the lives of women in Sudan is reproductive function and women lack autonomy in family decision-making; to some extent this may represent a violation of women rights [28]. Inheritance is patrilineal with primacy given to the husband, his family and male children, and men have authority over children. Although great value is traditionally placed on high fertility rates, some women overtly or covertly attempt to control their own fertility in the 21^st^ century [28,29]. It seems that in most sub-Saharan countries women’s roles in family planning and related decision-making processes are increasing, which offers empowerment to some extent with respect to maternity, pregnancy and their own health and well-being [29].

Decades of civil war in the Sudan forced many to leave the country or live in refugee camps located mostly in neighbouring countries. A large wave of immigration from Sudan to Canada began in the mid-1980s and continued until 2005. According to the 2006 census, approximately 13,000 Sudanese live in Canada [30]. Various other reports of informal population counts have claimed much higher figures between 40,000 - 80,000, with an “informed guesstimate” in 2009 of 35,000 - 40,000 [31]. Almost 30% of the immigrants from Sudan live in Alberta and the Sudanese living in Edmonton are mostly from South Sudan.

## Methods

This focus of this manuscript is to present research findings on the maternity experiences of women of Sudanese origin in an urban Canadian city. The data was captured during a larger mixed-methodological study aiming to increase understanding of how maternity services can better enable immigrant/minority women to have positive maternity experiences in urban and rural Alberta. The goals of the qualitative dimension of this work were to map out the experiences of immigrant women in maternity services and to capture the perspectives of healthcare professionals, policy makers, immigrant advocates and community representatives with respect to the challenges faced when attempting to optimize care for this population. The aim of this paper is to report on findings specific to the experiences of immigrant Sudanese women throughout the prenatal period and to explore the disparities, based on their ethnocultural beliefs, they may have experienced while receiving maternity care services in Alberta.

We used a focused ethnographic approach to explore the women’s experiences [32-35]. Focused ethnographies are characterized by *a)* a conceptual orientation of a single researcher, *b)* focusing on a discrete community or organization or social phenomenon, *c)* being problem-focused and context-specific, *d)* a limited number of participants, *e)* participants usually holding specific knowledge, *f)* episodic participation observation, and *g)* their use in development of health services. This methodology is suitable for exploring distinct groups of people within complex societies and uncovering underlying power relationships within a culture which may influence health care practices, opportunities and care-related decisions [36].

### Participants, recruitment and data collection

Purposive sampling was used to select women of Sudanese origin who had immigrated to Canada within the last 5 years and who were either pregnant and using health care services, or in the postnatal period up to one year following birth. We avoided recruiting women at the end of pregnancy or in the intrapartum and immediate postnatal periods (up to 4 weeks following delivery) since this is a sensitive time for both mother and baby; however if a woman expressed an interest in participation at this time then we were guided by their wish.

A cultural broker having Sudanese language skills and experience with research assisted in the recruitment, consent and interview processes. Recruitment was facilitated with the help of the Multicultural Health Brokers Co-operative; cultural brokers offer a unique service providing an interface between ethnocultural groups and the institutions of the recipient/host society, in this case, health services. Cultural brokers most frequently share the same first language and ethnocultural orientation of the women they are supporting, thus provide considerable support in respect of access to and navigation of health services. This benefits not only the women service users but also service providers since cultural brokers are able to offer providers ethnocultural insights. Focus group interviews (FGIs) were conducted by the first author in a community setting familiar to the women. Although the study documents (information letter and consent form) were written in English and the interviews were conducted in English, the cultural broker was present to serve as an interpreter for those women who had difficulties understanding the written documents and/or preferred to communicate in their own language. Based on a previous consultation exercise with key stakeholders in maternity care, a semi-structured topic guide was developed for use during the interviews. Written informed consent was obtained from all participants and the interviews were audio recorded, after obtaining the participants’ permission, and transcribed verbatim by a professional transcriptionist.

Ethical approval was granted for this study by the institutional review board (University of Alberta, Edmonton, Alberta) and informed consent to participation was obtained from all participants prior to data collection and after emphasizing the participants’ right to withdraw at any time.

### Analysis

Data were managed and analyzed with the aid of ATLAS.ti qualitative data analysis software (ATLAS.ti Scientific Software Development GmbH, Germany). Roper and Shapira’s [37] framework for analysis of ethnographic data was used because of: *a)* the relevance to ethnographic data, *b)* the clarity and transparency of the systematic approach, and *c)* the compatibility of the approach with computer-assisted qualitative data analysis software. The process of qualitative data analysis is characterized by identification and classification of data, with progression to abstractions and explanations of patterns of phenomenon within the cultural group. In this iterative process, preliminary interpretations are challenged and data are revisited in the light of further data collections and new insights into the data. Analytical steps included: *i)* coding for descriptive labels, *ii)* sorting for patterns, *iii)* identification of outliers or negative cases, *iv)* generation of themes, *v)* generalizing with constructs and theories, and *vi)* memoing and reflective remarks [37].

## Results

In total 12 immigrant Sudanese women (mean age 36.6) participated in two FGIs. The length of residence in Canada for the women was between a few months to 5 years and many had migrated from a country other than Sudan (Table 1). Analysis of the data revealed two major themes: personal agency and resistance to health practices, each with sub-themes.

**Table 1:** Demographic characteristics of the study sample

**Participant**	**Age**	**Marital status**	**Country of origin**	**Country immigrated from**	**Spoken languages**	**No. of children born outside Canada**	**No. of children born in Canada**
**1**	38	Married	Sudan	Egypt	Dinka, Arabic & Sudanese	2	3
**2**	41	Single	Sudan	Egypt	Balanda	2	1
**3**	45	Married	Sudan	Egypt	Arabic & English	2	1
**4**	36	Married	Sudan	Egypt	Arabic	0	2
**5**	31	Married	Sudan	Sudan	Balanda, Arabic	1	2
**6**	39	Single	Sudan	Sudan	Balanda & English	0	1
**7**	32	Married	Sudan	Egypt	Dinka & English	1	4
**8**	40	Married	Sudan	Egypt	Dinka & English	2	2
**9**	42	Divorced	Sudan	Lebanon	Arabic & English	1	0
**10**	25	Married	Sudan	Egypt	Arabic	0	1
**11**	38	Married	Sudan	Egypt	Arabic	1	0
**12**	33	Single	Sudan	Sudan	Nubia	0	2

### Personal agency

Personal agency refers to the notion that the person has the capacity to make her or his own choices and exercise autonomy over personal life events and circumstances. During labour and delivery, although the women are in extreme pain and may be able to freely express themselves, they may elect to remain silent as a result of social control. The participants in this study described how women in Sudan are encouraged to be strong; physical courage is admired and weakness is ridiculed. Interpreting their words, the expression of pain through screaming and exhibiting distress during labour and delivery were associated with weakness in their culture, and because dominant norms exert considerable pressure in respect of social control, the women often demonstrate stoicism during labour and delivery rather than a true expression of feelings.

P11: And we have something also in our tribe. If you’re going to have the baby you don’t have to cry and do this all kind of this funny stuff, because they’re going to sing a song about you that you’re a chicken or something…You can’t cry. You can’t do this funny face and this kind of stuff…You have to be strong for it.

The participants expressed that Sudanese women are expected to cook, clean, and generally look after their husband and children even when pregnant. Beyond physical strength, Sudanese women are expected to be mentally healthy. According to the participants, depression is rare in Sudan; parenthetically postpartum depression is apparently not viewed with much sympathy.

P11: No, it’s normal. They know if you are women you have to get pregnant, you have to have kids. We don’t have like, I’m depressed because I am pregnant or something, no.

This quote implies sociocultural values of having many children and can be seen as one of the reasons that some people do not encourage contraception. The other significant issue is the role of males in family planning. The participants noted that men are very reluctant to use condoms or other forms of birth control. Women often have to hide the fact that they are using birth control. This topic as well as the constraints of patriarchy was expanded upon by many of the participants.

#### Hidden contraception

Sudanese women are expected to have children but sometimes take contraceptives secretly, for example, while at their mother's house (presumably with the mother's knowledge) or by hiding pills in their clothing. As one participant said, you use birth control if you are "strong for yourself". This participant delayed having children for four to five year after marriage. This may be interpreted as personal agency, and an empowering dimension through which women gain some control over family planning decisions.

P11: Sometimes you’re going to be in big problem with your husband if - you know, some women they do it secretly…They say, I don’t know why I don’t have the babies, and he worry maybe sometimes. But if you want to discuss he will immediately tell you no.

One woman also noted that several people in her community have had tubal ligations without their husband’s knowledge.

P11: He said, “Oh, what’s happened?” She said, “Oh, maybe God don’t give me children. I don’t know.”

I2: So you use family planning without their knowledge?

P11: Sometimes some women use this one -

I1: Injection. Yes, yes.

#### Resistance to patriarchy

In Sudan’s patriarchal society, women have few rights and may be constrained by their husbands and communities. Especially in rural areas, the women are expected even when pregnant to do all of the housework, cooking, and childcare. After birth, women are expected to breastfeed and in many areas of Sudan there is no access to infant formula. A woman’s main reason for breastfeeding can vary and it is not usually only to nourish the baby but also for use as a natural form of birth control. While breastfeeding their children (the length of which varies by tribe and may be from 40 days to two years) Sudanese women are not usually able to have conjugal relations with their husbands; this situation is mostly influenced and guided by older females in the family such as the woman’s mother-in-law. The rationale seems to be that the health of the newborn child is paramount; therefore if the new mother is feeding the baby but gets pregnant again, the baby might lack attention and get sick and die leading the community to accuse the woman of not being a responsible parent. Since polygamy is socially accepted and legal in the Sudanese communities where these participants have origins, the husband may bring another “wife” into the household during this period. One participant laughed when stating that her brother had 11 wives. Thus, in rural Sudan, a woman who gives birth faces a terrible dilemma: in order to be a responsible parent, she may risk personal loss – her husband may find another woman/wife, without any right to complain.

P11: Your husband have to move out from the house [during and after baby delivery]. For one year, two years. When you are breastfeeding? Yeah…

P7: Also then they look for another woman. He will have two wives at home or three wives…You have to accept it because you have the baby, you stay there with your baby and husband will go enjoy…we don’t have like the right to speak out.

However, the socio-legal environment in Canada which promotes equality between the sexes coupled with the availability of contraception, seems to be changing the power dynamics between husbands and wives and conjugal relations within in the Sudanese community in our study population. These women are now able to resume sexual relations (“enjoy” their husbands) shortly after their delivery, without this decision being guided by mother in-laws in Sudan. Sudanese women in Canada can ask their husbands to take responsibility for family planning and to help with childcare and housework, even if this request may not garner a positive response. However, when the women assert their rights in Canada or refer to the Canadian cultural norm of fairness, this can sometimes be viewed by their husbands or in-laws as disrespectful and cause tension in the family.

### Resistance to health practices

The Sudanese women in the FGIs remarked that they had a great deal of experience with the birth process. Birth is seen as natural and a community event. Many of the participants indicated that even before having one's own child, one has much experience of caring for babies and small children including their younger siblings. Many of them had also been brought up (sometimes from the age of five years) seeing women give birth in their own communities in Sudan. Even when not related by family to the laboring woman, girls in the community are encouraged to participate by heating water, fetching cloths, and assisting with other small tasks. Community responsibility is modeled for them, and they also see the pain and reality associated with the birthing process.

P11: I don’t think so there is any different because they take it - because you already have the experience. Even when you are young you go always, if somebody have a baby, even young people, they will go there to watch… Yeah, to see how they deliver a baby

P7: Because, you know, when they come back they be there, it’s not because they want to be there. They send them [female child] with the stuff. Oh, you have to heat the water and bring it. You have to bring this one and this one.

Women in some villages in Sudan are unlikely to have doctors deliver their babies. Instead, they may have a midwife or even a traditional birth attendant who has had no formal training yet great experience with delivering babies. One woman’s mother was the local birthing specialist and delivered her daughter’s baby. These experiences may have led the women in the FGIs to conceptualize birth as a normal and natural process with which they have understanding and comfort. These conceptualizations may allow them to resist practices which they interpret as abnormal or unnatural, such as analgesia and delivery instrumentation.

#### Strength and knowledge in pain relief

In Canada, the participants still seem reluctant to have analgesia during labour (though they did report taking paracetamol/acetaminophen with codeine after a caesarean section). This may be because they are trying to be strong and stoical (as previously discussed); it may also relate to perceived negative side effects of the epidural anesthesia injections which were reported as bad headaches, losing hair, discomfort in the abdomen, and the risk of paralysis. One participant remarked that after initially refusing an epidural, she consented after three hard hours of active labour. As well, at least one other participant suggested that an epidural is not needed if the time of delivery is short (“less than 3 hours”).

#### Fear of caesarean sections

There appears to be a widespread belief for the participant women that women should not go to the hospital because the doctors will perform a caesarean section.

P11: Our people, they stay for a while because they say if you rush to the hospital they’re going to do for you c-section. We have that idea in our community. You have to wait until sometimes you deliver in the car or in the [ambulance].

These women’s reluctance to have caesarean sections may relate to their view of surgical births not being "natural" and thus representing medicalization of the process. Some participants described how they resisted their doctor’s advice to have a caesarean section. One participant said that she had to "try, try, try" to have a baby vaginally, and only had a caesarean section after several hours of labour:

P7: Yeah, but here they’re telling me, okay. I say, “You know what? I have to try, try, try,” but I couldn’t. Yeah, the last baby, the doctor told me, “Actually, you know, I don’t think you will have the normal delivery.” I said, “I have to try.” Okay, after I have the baby the second day he came over and he told me, “You know, I told you you don’t have a baby normal because you are small.

#### Resistance to other practices – delivery positions and relief for swelling

Even though the participants in this study generally complied with medical practices in Canada, there were many comments regarding an inconsistency between these practices and their own customs and perceptions. For example, Sudanese women had mixed feelings about delivery positions. In rural areas in Sudan, women are accustomed to kneeling or squatting to give birth. However, in urban hospitals in Sudan as well as hospitals in Canada, women are typically expected to deliver in the supine position. Participants reflected that a lying-down position is convenient because it is easier to clean up the blood and may be easier for the health professionals aiding the delivery. Although some participants had adapted to the supine position, other participants had a definite preference for the "rural" squatting position claiming this as easier and more comfortable. One participant stated, “Sitting this way, you get more pressure coming down, yeah, and help the baby to come out first”. Another participant indicated that for births in the hospital (where she was lying down), she was in pain and discomfort (“short of breath”).

P3: No, when you’re laying down it’s too much pain. If you walk it’s better.

Participants tend to follow their beliefs, even when they contradict evidence-based medicine recommended by Canadian health practitioners. A good example is that during the postpartum period they continue to put hot water on their swelling, despite the advice of nurses to use ice packs. As one participant said, "I have to do what I believe". This also illustrates the women’s agency - they are not cowed or intimidated by "advanced" Western health knowledge, and they will resist practices with which they do not feel comfortable.

P7: They told me that with c-section I have to put the ice with cloth but I go in the bathroom, take hot water with cloth. [laughter]…

I1: So do you think the hot water works on the wound?

P7: It’s my tradition. I believe in it, you know. . .I have to do what I believe.

#### Tradition related to hot and cold

There are also cultural norms associated with the consumption of food and beverages during maternity. As mentioned, in Sudanese villages, women in labour are not given any pain relief. However, great value is placed on "hot" things during labour, which are believed to reduce pain and speed delivery. Women drink hot liquids (water, tea), eat hot foods like porridge or soup, and are encouraged to take hot showers.

P2: Hot tea and hot drinks.

I1: Okay, so on the whole the hot things are thought to be good for labour?

P2: They mean to help you like help the baby go out.

Many of the Sudanese participants who had given birth in Canadian hospitals expressed dissatisfaction with the food. Although they appreciated being offered the option of a Halal menu, the women were often given cold food. According to their cultural beliefs, hot food after delivery is beneficial because it will reduce the blood in the stomach and benefit the baby. Some women said that they did not eat the hospital food but instead had food brought in by their friends and family.

P1: It’s mostly cold. Sometimes I don’t accept it. Sometimes I refuse it. Sometimes I take some like soup, hot soup I can, yeah, use it. I can eat it….

P3: Hot? Yeah. Because I know some people just after they have baby here in [Hospital], they never eat until they go home.

P11: For four day they don’t eat.

#### Bottle-feeding versus breast-feeding

Breastfeeding/bottle feeding the baby in the hospital arose as being an issue for some participants. As breastfeeding is associated with naturalness in Sudan, they are reluctant to engage in bottle feeding. Some of the women indicated that the hospital nurses did not ask about the new mother’s wish to breastfeed, or else ignored their expressed wishes to breastfeed. Participants also seemed a little wary of an apparent promotion of formula in the hospitals, as commented on at length by one participant who spoke through her own as well as shared experience.

P11: The only thing, sometimes they introduce bottle to the baby immediately, the nurses… They bring bottle, yeah. They don’t kind of like ask you to feed the baby…. Because I’ll sometimes go for labour support with the moms I see and they have that, they advertise about the formula. You couldn’t believe at the hospital. They have that all information on the formula and this one with this kind of good rich stuff on it, the formula, to give the moms interested to give it to the babies.

## Discussion

A society is not only a collection of individuals: norms, values, rules, beliefs, habits and cultural characteristics all shape society. ‘Beliefs’ are a major facet in medical behavioral sciences related to health and illness [26,38-40]. People do not necessarily lead their daily lives as instructed, but rather as they believe. “Beliefs are most closely associated, that is, with cultural accounts either of the unknowable or of mistaken understating of natural world” [26] p 20] thus they represent one of the main analytical units of culture.

There are many beliefs that may impact on pregnancy behaviors and attitudes about delivery and care of the infant. A study about cultural diversity and birth reports that some women believe that if they are not happy during pregnancy it can affect their baby’s characteristics [41]. Concurring with our findings, this study also revealed that women consider screaming and crying as shameful and wasted energy during the final stage of birth. Some believe that pain is obligatory [41]. The lack of obvious signals related to pain and distress may be misleading for some health care providers who are not familiar with their client’s cultural background. Outward display of pain in the time of pregnancy and labor is traditionally considered as “weakness” and cowardly throughout many African countries in addition to Sudan [42]. Furthermore, the support of male dominance of the family and the ideology of patriarchy within the sub-Saharan culture creates pregnancy and birth as a time for empowerment of the women, over their own and their child’s lives. During this period women desire to show their ability to manage their own life without showing any sign of weakness [25,27,29,41,42]. It is likely also the result of these cultural family norms and this power struggle faced by women that the women choose to exercise their ability to control fertility, yet do so in secrecy. Evidence shows that when males have strong dominance over fertility decisions, subservience is a main reason behind not using contraceptives outwardly, despite that in many cases the women use contraceptive secretly [43]. Using contraceptives is one such way for a woman to feel "strong for yourself". As mentioned in the results section, the Canadian law supports this right for women but since it may also cause family conflict many women prefer to do it secretly without permission from their husbands. Women usually decide to have fewer children for several reasons, because of pregnancy and its consequences, socioeconomic barriers and health based on the age of pregnancy [29].

The behavioral characteristics of individuals are shaped by sociocultural elements within their environment. Childbearing as one of the most important life events may be experienced differently in a different cultural context where different social beliefs exist [25]. Cultural differences between health care staff and patients and a lack of cultural awareness among patients and health care providers may lead to misunderstandings, which may place the health of mother and child at risk [9,10,20]. As Good [26] explored in his book entitled *Medical rationality and experience* signs and symptoms may be interpreted differently by physicians and patients. Moreover, because of cultural differences, terminology including the names of medical conditions can be translated differently.

Corin [44] refers to this cultural context as the cultural frame that involves a “thickening” of one’s understanding of the health phenomena. This cultural frame is a conceptual band of layers of factors consisting of *a)* the macrosocial construction of the determinants of health, *b)* sociocultural factors in the contexts of the home and host countries, and *c)* life history. All of the above-mentioned factors are important elements that interact to render meaning to a life event such as maternity, and to the reason behind resistance to health care practices among newcomer immigrant women. Many of this study’s participants preferred to follow the medical traditions associated with their country of origin. The Islamic, Greek, and Chinese medicines have their own unique principles and have been influential in many ethnocultural groups and societies through the spread of various historical conquerors and empires. For example, terms used frequently in Islamic, Eastern and Greek medicine are “warm” and “cold” [26,45,46]. On this basis, food and other entities are classified as having cooling and warming medicinal properties. Hot foods are considered to have high energy, such that after delivery when the woman’s body is weak and venerable to illness (cold), hot foods are provided for forty days to produce strength and increase the body’s heat to fight against the cold [26]. Avoiding cold extends beyond food to other entities such as bathing and use of cold compresses on areas of swelling. A study by Small et al. [47] revealed that even though bathing after delivery is recommended by health care providers, some immigrant women reject it because of beliefs about their potential negative effects on their body.

Beliefs and cultural values steer individual behaviors. Women who grow up in a different sociocultural context having different medical traditions usually face many challenges when in a new context and this may lead to misunderstandings and resistance to health care practices. During pregnancy, birth and after delivery many women rely on their beliefs formed by traditional medical practices and avoid procedures and activities which are thought to cause harm. For these women the pregnancy and labour is considered natural, whereby special attention is not required and interruption of this experience is unnecessary and may in fact harm themselves or their baby [15,25]. Many of our research findings are consistent with other work studying various cultures, indicating that women try to adjust their actions to align with different maternity customs (e.g. fertility) and health care approaches without ignoring their own existential concerns and beliefs. The following quote seems to sum up what the women are saying and how they experience maternity in new countries, taking into account their own beliefs and culture:

P7: You know, here for my opinion our culture here in Canada, some is good, some is not good, you know. For me I just take the good one and leave the one that I don’t like. Yeah, and put my culture that is good with me, I will put it beside it and the bad one I have to put it aside.

Although this paper provides information regarding the maternity and ethnocultural beliefs of our Sudanese women participants, we must acknowledge some of the limitations with regards to the generalizability of the findings. Firstly, the nature of qualitative research and having purposive sample selection does not allow us to generalize the findings to all Sudanese women in Canada. Secondly, we avoided selecting participants in their final stage of pregnancy or in the immediate postnatal period. This may have increased the risk of missing some valuable information and thus we recruited a large enough sample to enable us to answer our research question; this concept is mostly referred to as data saturation. Whilst our study sample may be considered a “vulnerable” population, we worked closely with a cultural broker (as described earlier) to minimize the potential of cultural incongruence with respect to our research procedures and protocols. We are also aware that Sudanese culture is not homogenous and there is diversity within this country especially with respect to geographical location (rural versus urban regions). Thus the findings of this paper are not necessarily representative of the entire maternity culture of Sudanese women in Sudan.

## Conclusion

The impact of globalization and mass immigration means that an essential requirement in many developed nation states is the existence of policies on multiculturalism and their extension to health care and maternity care provision. As Paul [48] said, “*If you wish to help the community improve its health, you must learn to think like the people of the community”* (p. 1). Seemingly, resistance to health care practices and recommendations can mislead health care providers should they not have cultural awareness, which can be observed as one of the important factors for providing appropriate care in multicultural societies. Maternity care and pregnancy and birth-related behaviors, which are highly based on general cultural beliefs and social norms, may be better understood and appreciated when health care providers take the opportunity to become more culturally sensitive.

## Competing interests

The authors declare that they have no competing interests.

## Authors’ contributions

GH led the conceptualization and design of the study, assisted with data collection, oversaw data analysis and revised the manuscript for intellectual content. JS contribute significantly to the interpretation of the data and drafted the manuscript. ZM, YC, and PP contributed to the conceptualization and design of the study and critically revised the manuscript. YC facilitated the recruitment and data collection of the participants. JP assisted with data collection and edited the manuscript. All authors have read and approved this version of the manuscript for publication.

## Pre-publication history

The pre-publication history for this paper can be accessed here:

http://www.biomedcentral.com/1471-2393/13/51/prepub
